# Innovations in repetitive transcranial magnetic stimulation: a review of clinical evidence and patient-reported outcomes for ExoTMS

**DOI:** 10.3389/fpsyt.2026.1850212

**Published:** 2026-07-20

**Authors:** David Pánek, Václav Gerla, Michelle Dees, Yael Halaas, JD McCoy, Louis Cady, Rakesh Nanda, Rudolph Eberwein, Henry Johnston, Nick Tvrdik, Georgine Nanos, Charmi Patel, Melinda Silva

**Affiliations:** 1 DP Neuro s.r.o., Prague, Czechia; 2 Czech Institute of Informatics, Robotics and Cybernetics (CIIRC), Czech Technical University, Prague, Czechia; 3 Luxury Psychiatry Clinic, Winter Garden, FL, United States; 4 Science & Beauty Associates, New York, NY, United States; 5 Contour Medical, Gilbert, AZ, United States; 6 Cady Wellness Institute, Newburgh, IN, United States; 7 Jiva Med Spa, Columbus, OH, United States; 8 A New You Wellness, Miami, FL, United States; 9 Aria Integrative Health, Denver, CO, United States; 10 Kind Health Group, Encinitas, CA, United States; 11 Minooka Healthcare Center, Chicago, IL, United States; 12 Silva MD Anti-Aging and Wellness, Chula Vista, CA, United States

**Keywords:** device design, ExoTMS, neurotechnology, noninvasive brain stimulation, transcranial magnetic stimulations

## Abstract

**Introduction:**

Transcranial magnetic stimulation (TMS) is a non-invasive neuromodulation technique with demonstrated efficacy in the treatment of multiple psychiatric and neurological disorders. ExoTMS represents an evolution in TMS system design, developed to optimize focused brain stimulation while enhancing patient comfort and operational efficiency. By leveraging established neuroplastic mechanisms in a more patient-centered format, ExoTMS may represent a scalable advancement when compared to conventional TMS systems. This review examines the technological characteristics, safety profile, and therapeutic implications of ExoTMS, with particular attention to patient-reported outcomes, treatment comfort, and safety.

**Methods:**

Eight studies were included, comprising 182 active-treated participants who received stimulation at effective intensity levels. Treatment protocols consisted of 4 to 6 sessions, with follow-up assessments conducted at 1 month and, in studies reporting extended follow-up, at 3 months post-treatment. Data collected included mapping time for coil positioning and setup, incidence of adverse events, and patient-reported outcomes. Subjective measures assessed treatment satisfaction and perceived comfort using questionnaires.

**Results:**

Patients reported high satisfaction with ExoTMS treatment, with improvements observed immediately post-treatment, and further increases at follow-up. Improvements were noted across multiple domains, including overall well-being, mood, perceived stress, and mental energy. Treatment tolerability was high, with 93.3% of participants reporting the therapy as comfortable and associated with minimal to no pain. No serious adverse events were reported.

**Conclusion:**

ExoTMS appears to be a well-tolerated TMS-based intervention characterized by favorable treatment comfort, minimal pain, and the absence of serious adverse events. Patient-reported outcomes suggest improvements in well-being and self-regulatory domains that persist beyond the immediate treatment period. Compared with conventional TMS systems, ExoTMS may offer practical advantages, including reduced treatment burden and improved clinical workflow efficiency.

## Introduction

Transcranial Magnetic Stimulation (TMS) is a non-invasive neuromodulation technique based on the principles of electromagnetic induction, first described by Faraday in the early 19th century, whereby time-varying magnetic fields induce electrical currents in neural tissue. Modern TMS was first implemented in 1985 by Barker and colleagues (University of Sheffield), who developed a device capable of delivering magnetic pulses that stimulate the human cortex without the discomfort with earlier electrical stimulation methods. Since its introduction, TMS has evolved from a neurophysiological research tool into a therapeutic modality. Throughout the 1990s and 2000s, the development of standardized safety guidelines and controlled clinical trials—particularly in major depressive disorder—supported its clinical adoption. In 2008, the U.S. Food and Drug Administration (FDA) cleared TMS for treatment-resistant major depressive disorder, and subsequent advances in coil design, targeting methodologies, and stimulation protocols have expanded its clinical and research applications (1).

Mechanistically, TMS operates through externally applied, rapidly changing magnetic fields that induce focal electric currents within cortical tissue. These induced currents depolarize neuronal membranes and modulate synaptic activity (2). Depending on stimulation parameters including frequency, intensity and patterns, TMS can produce inhibitory or excitatory effects on cortical excitability (3). Repeated stimulation sessions are believed to engage longer-lasting neuroplastic mechanisms analogous to long-term potentiation (LTP) and long-term depression (LTD), resulting in sustained modulation of functional connectivity within large-scale neural networks. Neuroimaging studies have demonstrated that stimulation of prefrontal regions influences cortico-limbic and fronto-striatal circuits involved in affect regulation, executive function, and stress responsiveness (4). Through these local and network-level effects, TMS is thought to normalize maladaptive neural activity patterns associated with psychiatric and neurological disorders (5).

Despite its favorable safety profile, TMS is often positioned clinically after unsuccessful pharmacological and psychotherapeutic interventions. Pharmacological treatments, although widely prescribed, are frequently associated with undesirable side effects including weight gain, cognitive dulling, sedation, sexual dysfunction, and mood instability, which may lead to poor adherence and increased relapse risk. (6, 7) Psychotherapy remains a cornerstone of care; however, meaningful symptom improvement often requires 12–20 sessions and depends substantially on patient engagement and therapeutic alliance. (8) These limitations highlight the need for alternative or adjunctive modalities that offer effective symptom relief with improved tolerability.

TMS is generally regarded as safe and well tolerated, although mild to moderate scalp discomfort may occur depending on stimulation parameters and device design. (9) Current clinical protocols typically recommend 20–30 treatment sessions for optimal outcomes. (10, 11) Beyond total session number, clinical efficiency is influenced by workflow-related factors, particularly the accuracy and reproducibility of coil positioning. Precise applicator placement is essential for consistent stimulation of targeted cortical regions; however, traditional systems often require 11–30 minutes for preparation and alignment due to manual adjustments and operator-dependent fine-tuning. (12) These setup demands may reduce clinic throughput, increase operational costs, and introduce inter-session variability.

In response to these practical and operational challenges, newer TMS platforms have been developed to improve treatment efficiency, scalability, and user experience. We hypothesize that the ExoTMS platform’s integrated navigation and condensed protocol structure can achieve clinical satisfaction and operational efficiency comparable to conventional systems while significantly reducing setup time and operator-dependent variability.

### ExoTMS technology

ExoTMS represents an evolution of conventional TMS systems design, developed to optimize stimulation delivery, enhance patient comfort, and improve operational efficiency while preserving established neuromodulatory mechanism.

From a neurophysiological perspective, electromagnetic stimulation seeks to restore impaired neural network function through activity-dependent plasticity. Repeated stimulation is hypothesized to reinforce weakened synaptic pathways and strengthen functional connectivity, thereby counteracting disrupted neuronal communication commonly observed in neuropsychiatric disorders (13). These mechanisms are consistent with contemporary network-based models suggesting that TMS promotes large-scale neural reorganization and enhances cortical-subcortical connectivity associated with clinical improvement (14).

**Figure 1:**
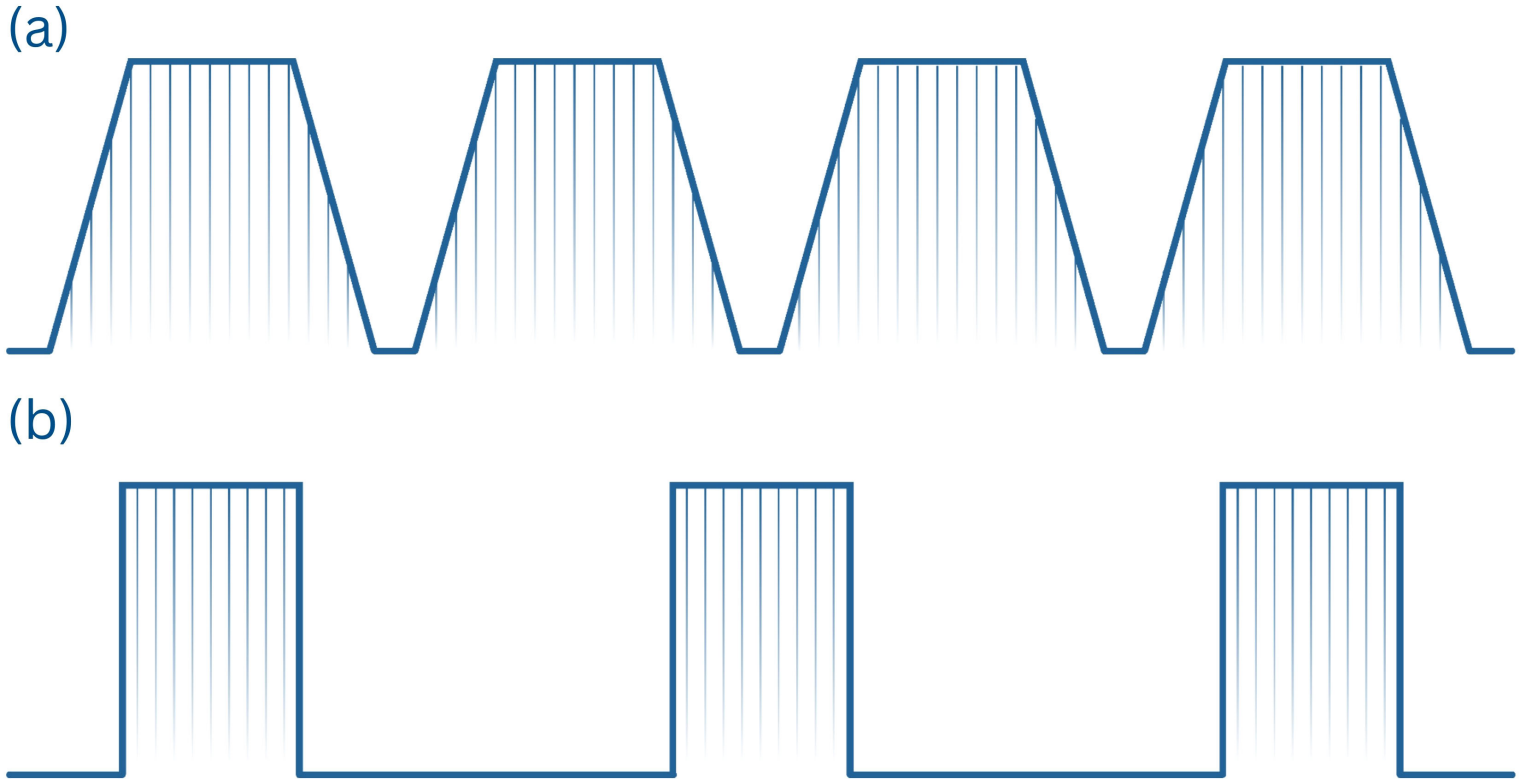
ExoTMS ramp-up stimulation pulse **(a)** and the standard transcranial magnetic stimulation pulse **(b)**.

To enhance patient comfort, the system incorporates a ramp-up stimulation mode (see **Figure 1**), in which stimulation intensity is gradually increased at the initiation of each session (15). Progressive titration of intensity has been shown to reduce sensory discomfort and improve tolerability during repetitive stimulation (16).

The device further employs an innovative air-cooling system designed to prevent coil overheating during stimulation (17). Effective thermal regulation shortens treatment time and enables sustained operation during high-frequency protocols without performance degradation (18).

The applicator utilizes a parallel wiring architecture with dual conductive cores to reduce resistive energy loss during pulse transmission and improve magnetic field efficiency (19). Optimized coil engineering, including reduced electrical resistance and improved current flow dynamics, is closely associated with more stable field output and enhanced precision of cortical targeting (20).

The ExoTMS system integrates multiple technological & design innovations intended to address key limitations of conventional TMS devices. A distinctive feature of ExoTMS is its unique double-core stimulation coil, capable of delivering a substantially higher number of pulses per therapy session (21). In combination with active cooling system, this architecture permits extended operation without prolonged inter-session waiting periods, thereby supporting improved clinical throughput and workflow efficiency. In addition, the ability to deliver a higher pulse dose within a single session may also allow for protocol optimization, including potential reductions in total treatment sessions, while maintaining established therapeutic principles.

An additional engineering consideration of the ExoTMS system is the waveform of the stimulation pulse. ExoTMS utilizes a pulse with a gradual rising edge, which has been associated with improved patient-perceived comfort. In contrast, many conventional TMS systems utilize rectangular pulse waveforms that may be associated with a greater sensory discomfort or pain during stimulation. Improved patient comfort may enhance treatment tolerability and adherence, particularly over longer treatment courses (22).

Furthermore, the ExoTMS applicator incorporates a navigation-guided coil positioning system with defined orientation and target markers (23). This design reduces neuronavigation time and enables rapid, accurate applicator placement while minimizing reliance on repeated manual adjustments and limiting operator-dependent variability. As a result, setup time is reduced and session-to-session reproducibility of stimulation delivery is enhanced, supporting both workflow efficiency and consistency of treatment.

## Aim

This review evaluates the clinical feasibility and patient-centered outcomes of the novel ExoTMS technology. By synthesizing available clinical data from active-treated cohorts, the study characterizes the tolerability profile, treatment experience, and operational efficiency associated with this evolution in TMS delivery.

## Methods

This review synthesizes data from eight interventional clinical studies evaluating the ExoTMS technology across multiple research topics. Five studies were conducted in Europe (four in the Czech Republic and one in Bulgaria), and three were conducted in the United States. All studies received approval from the respective local ethics committees prior to initiation (see **Table 1** for an overview of the included studies).

**Table 1:** Comprehensive overview of the eight core studies included in this review. By listing the author, year, study design, sample size, and specific outcomes for each investigation, this table enables readers to systematically verify the primary evidence base discussed in the manuscript.

Author	Year	Study design	Sample size	Outcomes
Toni Slavchev, MD, David Pánek, MD, PhD	2023	Binge Eating EU	38	4.2 lbs average weight reduction, 74% showed no binge eating symptoms after the last treatment
Michelle Dees, MD	2023	Mental Well-being	33	88% showed improved well-being scores
Monika Klírová, MD, PhD	2023	Food Craving	23	3.3 lbs average weight reduction, FCQ-T males improvement 36%, females 34% after 1-month follow up
Louis Cady, MD	2024	Willpower/Food Craving	39	76% of patients felt more control of their everyday diet and 74% felt mentally stronger
Georgine Nanos, MD	2024	Sleep/Stress	31	86% reported improved sleep quality and 79% reported waking up more refreshed
David Pánek, MD, PhD, Václav Gerla, MSc, PhD	2025	EEG Brain Activity	12	The navigation system delivers highly accurate placement
Monika Klírová, MD, PhD	2025	fMRI Food Craving	6	47% reduced perceived food palatability

Across the included studies, designs ranged from single-center or multicenter, open-label, single-arm interventional trials to multicenter, two-arm, sham-controlled, single-blinded interventional trials. A total of 182 participants who received active stimulation were included in the pooled analysis. Demographic data were available for the majority of participants; among these, 52 were male and 130 were female, with ages ranging from 22 to 78 years.

Participants across the eight included studies were treated for a variety of clinical and sub-clinical indications, primarily focusing on metabolic and psychological health. These included binge-eating behaviors (24), food cravings (25–27), and diminished psychological well-being (28, 29) characterized by heightened stress and sleep disturbances. (30).

Depending on study protocol and indication, subjects underwent between 4–6 ExoTMS treatment sessions (EXOMIND, BTL Industries Inc., Boston, MA), administered at intervals of 2–10 days. Each treatment session lasted approximately 25 minutes, with stimulation parameters individualized according to motor threshold (MT) and patient tolerability.

During treatment sessions, subjects were positioned in a semi-reclined posture, and the applicator was placed over the left Dorso Lateral Prefrontal Cortex (DLPFC). Target localization was performed using the 5-cm method, involving identification of the motor cortex hotspot followed by measurement 5 cm anteriorly along the parasagittal plane. Stimulation frequencies ranged from 12 to 18 Hz, with 2-second train durations and 5-second inter-train intervals. Stimulation intensity was set between 70% and 100% of each participant’s individual motor threshold. .

To enable pooled analysis, consistent outcome measures were identified across studies. These included the Subject Satisfaction Questionnaire (SSQ), Therapy Comfort Questionnaire (TCQ), and mapping time required for motor threshold determination. Standardized assessment timepoints included baseline, post-treatment evaluation, and follow-up visits, depending on the study design.

Follow-up assessments were conducted at either 1 month or 3 months post-treatment, depending on the individual study protocol. Operational feasibility was assessed using mapping time, defined as the duration required to determine the motor threshold and position the applicator at the appropriate stimulation target prior to treatment initiation. Mapping time was recorded at two participating sites.

Subjective satisfaction was quantified using the Subject Satisfaction Questionnaire (SSQ), which included a core set of standardized questions common across studies, supplemented by indication-specific questions. Responses were rated on a 5-point Likert scale (1 = strongly disagree to 5 = strongly agree). Satisfaction outcomes were summarized as the proportion of participants reporting agreement or strong agreement with each statement.

Treatment tolerability was specifically isolated using the Therapy Comfort Questionnaire (TCQ), a single-item metric assessing agreement with the statement “I found the treatment comfortable” To provide an objective anchor for these subjective reports, pain intensity was recorded using the Numerical Analog Scale (NAS). This validated, 10-point scale ranging from 0 (no pain) to 10 (worst pain possible). Safety and tolerability were further evaluated through systematic recording of adverse events (AEs) throughout the study period.

Outcome data were summarized descriptively across studies, focusing on changes observed in active-treated patients.

## Results

### Subject satisfaction questionnaire

The Subject Satisfaction Questionnaire (SSQ) was administered across all included studies. Because certain items differed slightly between study protocols, only those questions that were consistently included across studies were analyzed (**Table 2**). Overall satisfaction with treatment results was reported by 77.0% of participants immediately post-treatment, increasing to 83.1% at the 1-month follow-up. Improvement in overall well-being was reported by 65.7% of participants immediately following treatment, increasing to 73.8% at 1 month. At the 3-month follow-up, 68.8% of participants continued to report improved well-being. Perceived stress reduction was reported by 72.6% of participants immediately post-treatment and remained stable at 72.6% at 1 month. At the 3-month follow-up, 78.1% of participants reported reduced stress. .

**Table 2:** The subject satisfaction questionnaire, percentages represent the percentage of subjects in agreement with the statement (n= indicate the number of studies in which the given question was included).

No.	Statement	After	1M	3M
Q1 (n=4)	Well-being has improved	65.7%	73.8%	68.8%
Q2 (n=6)	I am satisfied with the treatment results	77%	83.1%	65.8%
Q3 (n=3)	I feel the treatments have improved my mood	81.7%	77.2%	71.0%
Q4 (n=2)	I feel more motivated	72%	62.9%	64.1%
Q5 (n=2)	Handle challenging situations better	64.8%	67.1%	64.7%
Q6 (n=2)	I feel calmer after the treatment	78.6%	75.8%	75.0%
Q7 (n=2)	I have more mental energy after the treatment	77.3%	72.4%	75.0%
Q8 (n=3)	I feel less stressed after the treatment	72.6%	72.6%	78.1%

### Treatment comfort questionnaire

Overall treatment comfort was high, with 93.3% of active subjects reporting the treatment as comfortable. Correspondingly, participants reported minimal to no pain, with a mean Numeric Rating Scale (NRS) score of 0.7.

### Mapping time

The efficiency of the clinical workflow was evaluated by the duration required for motor threshold (MT) determination. This metric was recorded across two clinical sites involving a cohort of 83 patients. Within this group, the mean mapping time—defined as the interval from initial coil placement to the identification of the individual’s effective stimulation intensity-was 2.7 ± 1.58 minutes. The observed mapping times ranged from 1 to 7 minutes, suggesting a consistent setup process across different clinical environments.

### Adverse events

Across all study sites, no serious adverse events were reported. All reported events were mild and transient. The most frequently reported event was mild headache. Other isolated events included transient sensory discomfort, somnolence, nausea, dizziness, subjective fatigue, and temporary scalp pain. The overall safety findings were consistent with established data on TMS tolerability.

## Discussion

### Patient-centered outcomes and clinical adherence

The present review highlights several clinically and operationally relevant features of the ExoTMS platform that extend beyond conventional efficacy metrics. Across studies, patient-reported outcomes demonstrated consistently high levels of satisfaction and favorable tolerability. Improvements assessed by the Subject Satisfaction Questionnaire (SSQ) included domains of mental well-being, mood, motivation, mental energy, and perceived stress, with overall satisfaction increasing from 77% immediately post-treatment to 83.1% at one-month follow-up. Although partial attenuation was observed in some domains over time, several measures remained stable at three months, suggesting persistence of perceived therapeutic benefit.

Patient-reported satisfaction carries clinical relevance beyond subjective appraisal. In neuromodulation therapies requiring repeated visits, perceived comfort and treatment acceptability may directly influence adherence, engagement in follow-up care, and willingness to pursue maintenance sessions when clinically indicated. Positive treatment experiences have been associated with improved compliance and reduced attrition across various therapeutic modalities. Accordingly, the high proportion of participants describing ExoTMS as comfortable (93.3%), with minimal to no pain, represents a meaningful practical advantage that may support treatment continuity and long-term engagement.

The favorable safety and comfort profile observed here aligns with broader industry trends identified in recent longitudinal analyses. For instance, a 10-year analysis of the MAUDE database highlights that continuous technological advancements in TMS delivery have been pivotal in maintaining low adverse event rates while improving the patient experience. This suggests that the ExoTMS architecture is part of a necessary evolution toward more patient-centric neuromodulation (31).

### The shift toward accelerated protocols

The reduced number of treatment sessions observed in the studied protocols may further contribute to this potential advantage. Conventional TMS protocols typically involve 20–30 sessions administered over several weeks, which can impose logistical and economic burdens and potentially lead to treatment discontinuation. Similarly, long-term pharmacotherapy requires sustained adherence and is frequently limited by side effects, increasing relapse risk. In contrast, ExoTMS protocols in the reviewed studies involved only 4–6 treatment sessions while delivering a high cumulative number of pulses per session. The ability to achieve clinically meaningful improvements within a condensed treatment schedule may reduce overall treatment burden and improve feasibility, particularly in populations for whom extended regimens are impractical.

The move toward a condensed 4–6 session schedule reflects a significant shift in the field toward ‘accelerated’ protocols. Recent high-level evidence, such as the trial by Ramos et al. (2025), has demonstrated that intensive, accelerated stimulation schedules are not only feasible but highly effective in challenging populations like those with treatment-resistant depression. By achieving clinical impact in a fraction of the time required by conventional protocols, ExoTMS addresses the primary barriers of cost and time-commitment that often lead to patient attrition (32).

### Balancing intensity and tolerability

Importantly, the delivery of a high number of pulses did not compromise comfort. The incorporation of ramp-up pulse delivery and stimulation intensities tailored between 70% and 100% of individual motor threshold may mitigate abrupt sensory discomfort typically associated with high-frequency stimulation. In the broader TMS literature, scalp pain and headache are frequently reported and may contribute to early discontinuation in some patients. The favorable comfort profile observed in this review suggests that stimulation dynamics and device architecture play a critical role in shaping patient experience, beyond pulse count alone.

### Mapping time

Operational efficiency represents critical dimension in real-world clinical implementation. The short average mapping time of 2.7 minutes (n = 83) observed across two studies reflects a streamlined motor threshold determination process, reducing the duration patients spend in preparatory positioning and minimizing procedural burden. Compared with previously reported preparation times and labor-intensive setup for conventional TMS platforms, this duration is notably shorter. For example, systems utilizing H-coils often involve a 20–30-minute preliminary adjustment phase during which motor threshold mapping and coil positioning are calibrated (33). Similarly, conventional figure-of-eight coil systems commonly require approximately 20–30 minutes for initial setup without positioning aids, with additional time required when accessory caps or guidance tools are used (34). These procedures often involve repeated measurements and operator-dependent fine-tuning, contributing to extended preparation times and increased workflow complexity.

From a systems perspective, simplified applicator positioning may decrease operator-dependent variability and reduce the potential for alignment errors, thereby supporting improved reproducibility across sessions. Notably, ExoTMS system does not require neuronavigation for standard clinical implementation, distinguishing it from several conventional platforms that rely on landmark-based measurements or navigation-assisted positioning to ensure accurate targeting. At the same time, ExoTMS remains fully compatible with external neuronavigation systems, allowing integration of MRI-guided or functional targeting approaches when desired. This flexibility enables clinicians to adapt implementation according to infrastructure, clinical preference, and patient-specific needs (35, 36).

Collectively, shorter setup times may enhance clinical throughput, reduce staff workload, and limit opportunities for procedural inconsistencies—factors that are essential for scalability, cost-efficiency, and broader accessibility of neuromodulation therapies.

### Safety

Across the included studies, ExoTMS treatment demonstrated a favorable safety and tolerability profile. No serious adverse events were reported among participants receiving active treatments, and all observed adverse events were mild and transient in nature. Reported effects were primarily expected stimulation-related symptoms, such as mild headache or transient scalp discomfort, and did not require clinical intervention. These findings are consistent with established TMS safety profiles. Although conventional TMS is generally regarded as safe, published data indicate that headache and scalp discomfort may occur in a substantial proportion of patients (37). The comparatively low adverse event burden observed in this analysis suggests that ExoTMS maintains the recognized safety profile of TMS while potentially supporting improved tolerability. However, direct comparative trials would be required to confirm this observation.

Although certain neurological conditions have traditionally been considered as relative contraindications or warrant caution in the application of transcranial magnetic stimulation, accumulating evidence indicates that TMS can be administered safely in carefully selected populations. The safety findings observed in the present review are therefore consistent with, and supported by, a broader body of literature demonstrating that TMS-based interventions can be applied safely across diverse clinical populations when appropriate screening and protocol parameters are used. Importantly, the absence of serious adverse events and the low incidence of mild transient symptoms further reinforce the tolerability of the ExoTMS platform.

### Broader clinical implications

Beyond safety, the convergence of improvements across domains of well-being, self-regulation, mood, and perceived stress suggests that ExoTMS may have applicability across a broader range of indications. Emerging exploratory investigations have begun to assess its potential use in populations experiencing fatigue, postpartum and post menopause depression (38). While these applications remain preliminary, the observed pattern of patient-reported benefit across heterogeneous cohorts supports the possibility that ExoTMS may exert modulatory effects on shared neural networks involved in executive control, affect regulation, and stress responsiveness.

### Strengths, limitations, and potential biases

A principal strength of this review is the integration of clinical and patient-reported outcomes across multiple interventional studies evaluating the ExoTMS system, encompassing a relatively large cohort of actively treated participants across several geographical sites. The inclusion of patient-centered measures, including treatment comfort, satisfaction, and tolerability, provides clinically relevant insights into real-world treatment experience. In addition, the inclusion of operational metrics, such as mapping time, offers practical information relevant to clinical implementation.

Several limitations warrant consideration. The reviewed studies varied in design and included heterogeneous populations, and not all studies incorporated control or sham conditions, thereby limiting causal inference of observed effects. Outcomes were primarily self-reported, introducing potential response bias. Furthermore, follow-up durations were relatively short, restricting assessment of long-term durability. Additionally, certain feasibility measures, including mapping time, were available from only a subset of sites. Future randomized controlled trials with standardized outcome measures, longer follow-up periods, and direct comparisons to conventional TMS platforms are necessary to more fully characterize the clinical and operational advantages of ExoTMS technology.

## Conclusion

Available clinical evidence suggests that ExoTMS delivers effective, well-tolerated neuromodulation with a positive treatment experience, supporting its utility where medication is insufficient or poorly tolerated. The observed improvements across multiple domains, achieved within 4–6 treatment sessions, indicate potential feasibility of shorter treatment protocols relative to conventional rTMS regimens. These preliminary findings support further controlled studies to more definitively characterize clinical efficacy, durability of response, and comparative effectiveness within the broader landscape of non-invasive brain stimulation.
